# Does the Person-Centered Care Model Support the Needs of Long-Term Care Residents With Serious Mental Illness and Intellectual and Developmental Disabilities?

**DOI:** 10.3389/fpsyt.2021.704764

**Published:** 2021-11-12

**Authors:** Elizabeth P. Howard, Lynn Martin, George A. Heckman, John N. Morris

**Affiliations:** ^1^Connell School of Nursing, Boston College, Newton, MA, United States; ^2^Hebrew SeniorLife, The Hinda and Arthur Marcus Institute for Aging Research, Boston, MA, United States; ^3^Department of Health Sciences, Lakehead University, Thunder Bay, ON, Canada; ^4^Centre for Education and Research on Aging and Health, Thunder Bay, ON, Canada; ^5^School of Public Health and Health Systems, University of Waterloo, Waterloo, ON, Canada; ^6^Schlegel-University of Waterloo Research Institute for Aging, Waterloo, ON, Canada

**Keywords:** person-centered care, nursing homes, interRAI, serious mental illness, intellectual and developmental disabilities

## Abstract

Person-centered care approaches continue to evolve in long-term care (LTC). At the same time, these settings have faced increased challenges due to a more diverse and complex population, including persons with intellectual and developmental disabilities (IDD) and serious mental illness (SMI). This study examined the mental, social, and physical wellbeing of residents with different diagnoses, within a person-centered care model. It was hypothesized that individual wellbeing would be comparable among all residents, regardless of primary diagnosis. The study cohort was drawn from all admissions to long-term care facilities in the USA from 2011 to 2013. Data are based on admission, 3 and 6 month follow-up Minimum Data Set (MDS) 3.0 assessments. The groups examined included: schizophrenia, other psychotic disorders, IDD, dementia, and all others (i.e., none of the above diagnoses). The wellbeing outcomes were depression (mental), pain (physical), and behaviors (social). All residents experienced improvements in pain and depression, though the group without the examined diagnoses experienced the greatest gains. Behaviors were most prevalent among those with psychotic disorders; though marked improvements were noted over time. Improvement also was noted among persons with dementia. Behavior worsened over time for the three other groups. In particular, those with IDD experienced the highest level of worsening at 3-month follow-up, and continued to worsen. The results suggest person-centered care in US nursing homes provides the necessary foundation to promote mental and physical wellbeing in persons with complex needs, but less so for social wellbeing.

## Introduction

As health care systems struggle to evolve and transform to meet changing needs of the population, long term care settings face increased challenges to support an increasingly diverse and frail adult population—many, but by no means all of whom, can be classified as elderly. Contributing to the diversity of residents served is the unfolding impact of providing care to persons with intellectual and developmental disabilities (IDD) and serious mental illness (SMI), persons who previously (and in some cases, still are) were served in specialized institutions. As individuals with SMI age, their needs are less likely to be met through a combination of family support and community-based programs. Additionally, the life expectancy of persons with IDD continues to increase; although notably, premature aging occurs in this population with the designation of “old” occurring at a younger age than the general population (1, 2). Consequently, there is a growing prevalence of long-term care residents with mood and behavior issues that may challenge implementation of therapeutic interventions designed for others (3, 4).

In the US, more than three decades have passed since there were major reforms within LTC settings stemming from nursing care being deemed inadequate for meeting the needs of the older adult population. Central to the reform was a major shift from a nursing and institutional based approach to that of a person-centered culture. Formerly known as the National Citizen's Coalition for Nursing Home Reform, the National Consumer Voice for Quality Long-term Care is often credited with initial reform efforts, focusing on the rights of residents (5). Building upon the recommendations of the Coalition, the Institute of Medicine issued a report in 1986 that advocated for a home-like atmosphere and improved quality of care in an attempt to address the needs of the individuals in nursing home (6). The Omnibus Budget Reconciliation Act of 1987 included the Nursing Home Reform Act that required provision of person-centered care that promoted individual well-being (7, 8). While the term well-being is widely used, there exists no universally agreed-upon definition, resulting in it being understood and measured in different ways. A recent systematic review identified 99 different measures of wellbeing designed for adults (18 years or more) that touched on nearly 200 different dimensions (9). They noted that definitions and measures most commonly used included consideration of mental, social, and physical wellbeing.

### Admission to LTC

Long-term care settings commonly are regarded as placements for older individuals with cognitive and/or functional losses necessitating assistance with daily care activities. Historically, there has been a reluctance to accept persons with SMI into long-term care because of fear of mental illness and violence (10) as well as a concern for the limited availability of gero-psychiatric consultations within these settings (11). Yet, as time has passed a significant and increasing proportion of adults entering long-term care facilities in the US have prior or existing mental health diagnoses and IDD associated with mood and behavioral issues, creating complex challenges to optimal care provision. Published reports internationally cite that up to 8% of the nursing home population has a chronic mental health illness (12). A recent Canadian study found 40% of residents had SMI (13) and in the US, reports indicate up to 50% of nursing home residents have a diagnosed mental illness (3, 4, 14).

Individuals with a SMI diagnosis tend to enter nursing homes at a younger age despite having lower rates of cognitive impairment, and functional limitations (15–19). Similarly, while representing a small proportion of the LTC population, adults with IDD are, in fact, over-represented in the setting. A recent study found that rates of admission to LTC was up to nine times higher among those with IDD compared to the general population; this study also showed they were, on average, 25 years younger when admitted (19, 20).

### Stigma and SMI and IDD

Historically, negative societal attitudes toward deinstitutionalization have adversely affected persons with SMI (21) with self-stigma or the negative perception of self being not the least withstanding (22). Both public and self-stigma contribute to common behavioral manifestations of disinterest, distraction, avoidance, fear, shame and withdrawal (23). Because of increased vulnerability of LTC residents with SMI, they are more likely to exhibit aggressive and behavioral disturbances (23).

Just as much as in the general population, living and aging in the community is a high priority for individuals with IDD and their caregivers (24–26). In fact, given long-standing efforts to move away from institutional settings and toward community living in this field, remaining in the community becomes even more important. A recent study of professionals in the field of IDD reported that persons with IDD experience stigmatization within LTC by both the professionals who work there, and by other residents (27). Admission to LTC was itself stigmatized with many suggesting that it should be considered only as a last resort, upon the failure of community-based services to meet the needs of adults with IDD and their caregivers. For many, and especially older adults with IDD, admission to LTC represents a form of re-institutionalization.

### Physical Health, Mental Health, and Behavior

LTC residents with SMI often carry the dual burden of mental and physical co-morbidities (28). Older adults with SMI face challenges with mobility and functional capacity and experience higher rates of mortality and illness (29, 30). When compared with persons living with family, LTC residents with schizophrenia are more likely to have a decreased quality of life (31). Outside the United States, across nursing homes in the Netherlands, van den Brink et al. (32) found that 8 months after admission, those with mental-physical multimorbidity demonstrated increased hyperactivity, irritability occurring most commonly, and also a high persistence of depression. Additionally, residents in nursing homes in the Netherlands experiencing depression, had decreased well-being (33).

Adults with IDD of all ages also have more health conditions compared to the general population, a trend that continues throughout life. While Marengoni and colleagues (34) estimated that about half of older adults in the general population experience multi-morbidity, McCarron and colleagues (35) reported that about 71% of adults with IDD aged 40 years or higher had multi-morbidity, and about 80% of those 50 years of age or higher (36). Not surprisingly, adults with IDD have higher rates of health care service utilization overall (37). A group with many challenges, but a good one in which to test whether the Centers for Medicare and Medicaid Services (CMS) mandated personal model of care can result in outcomes that parallel those of other groups under study.

Nursing home residents with SMI may present with various behaviors that challenge including verbal aggression, repeated requests for attention, delusions, irritability and apathy (38). Among cognitively intact nursing home residents, depression, anxiety and a lack of social contact contribute to reduced quality of life and increased suffering (39, 40). The overall effects of these manifestations are associated with a reduced level of well-being (41–43).

In addition to impairments in cognition, social skills, and functioning, adults with IDD are also at increased risk for mental illness and behaviors that challenge (e.g., aggression, self-injury, destruction, pica) (37, 44). Schizophrenia and other psychotic disorders, for example, are prevalent in ~5–10% of adults with IDD (45), though many have suggested that this condition may be over-diagnosed (46). Others have reported on the higher prevalence of several other mental health conditions among adults with IDD, such as depression (47) and dementia (48, 49). The prevalence of mental health conditions in this population varies widely based on the setting (e.g., community, institution) and sample (e.g., age, type of IDD, level of IDD severity), but it is thought to be up to five times higher than in the general population (50). For their part, behaviors are among the most widely studied issues in this field (51); such behaviors have a tremendous effect on the quality of life of individuals (52) and contribute to the complexity of supports (3, 4, 53). Similar to prevalence of mental health issues, the prevalence of behaviors is difficult to determine—again due to study setting and populations studied, and also to the definition of “challenging” used (54). Consequently, prevalence ranges from 14 to 67% (55).

The prevalence of mental, physical, and multiple comorbidities among long term care residents requires the CMS mandated emphasis on person-centered care with a focus on symptoms and associated behaviors rather than diagnosis, to better address their needs and improve well-being (56).

The primary purpose of this paper is to examine elements of mental, social, and physical wellbeing among persons living in long-term care, and compare them among those with dementia, IDD, and SMI. Specifically, mood, behavior, and pain will be examined at admission (i.e., baseline), and at 3 and 6 month follow-up. The changes in measures of well-being are compared over time, and the differential subgroup effects over those time periods examined. It is hypothesized that with implementation of the person-centered care model, individual wellbeing will not differ among subgroups (i.e., dementia, SMI, and IDD).

## Materials and Methods

Anonymized data are held on a secure server at the Marcus Institute for Aging Research at Hebrew SeniorLife in Boston, Massachusetts. The data are analyzed subsequent to an ethics board approval through that institution.

### Data and Study Population

The study cohort was drawn from all admissions to US LTC facilities in the years 2011–2013; there were 2,286,724 admission assessments. The number of cases declined for the 3-month (*n* = 1,752,344) and 6-month (*n* = 1,093,890) subsets of data. Over the study time period, loss of subjects was due to multiple factors including hospital admissions, transfer to another LTC facility, and death.

The new admissions sample was grouped according to the following diagnoses, recorded in the assessment: schizophrenia (i.e., schizoaffective and schizophreniform disorders), mental health disorder other than schizophrenia, intellectual and developmental disability (i.e., Down syndrome, autism, epilepsy, other organic condition related to IDD, IDD with no organic condition), dementia (i.e., Alzheimer's disease, vascular or multi-infarct dementia, mixed dementia, frontotemporal dementia, r/t stroke, Parkinson's disease dementia, and Creutzfeldt-Jakob disease dementia; note: without schizophrenia, psychotic disorder, or IDD diagnosis), and all others (i.e., none of the above diagnoses).

### Instrument

Data used in the secondary analysis come from the Minimum Data Set (MDS) 3.0 (57). Containing over 300 items targeting the key domains of personal information, cognition, function, diagnoses, physical and mental health, behavior, service use, the MDS is the primary screening and comprehensive geriatric assessment of health status for patients in LTC. The MDS is completed by trained facility clinical staff at admission and quarterly thereafter, for the duration of the person's stay in LTC, as mandated by the Centers for Medicare and Medicaid in the US.

### Variables

Three primary outcomes included within the MDS assessment system were examined: depression, pain, and behaviors that challenge. The presence of depression was measured using the PHQ-9 tool contained in the MDS 3.0 (58, 59). It assesses mood status over the past 14 days, and uses a scale of 0 to 3 to score each of its nine items (0 = “not at all” to 3 = “nearly all the time”). Total scores range from 0 to 27 with higher scores representing more severe depression. Scores 0–4 were categorized as no depression and scores 5 or higher represented the continuum of mild to severe depression (58). The assessment item on pain frequency was used to identify the presence of pain. Presence of pain was indicated if the trained assessor scored the person as having any sign of pain.

Behaviors that challenge were defined as present if any of the following was exhibited by the individual: wandering, physical behaviors directed toward others, verbal behavior directed toward others, self-injuring behaviors (e.g., hitting or scratching self), socially inappropriate behaviors (e.g., rummaging, public sexual acts, disrobing in public), and disruptive behaviors (e.g., throwing or smearing food or bodily waste, screaming, disruptive sounds).

### Analysis

Descriptive statistics (%, mean, standard deviation) are used to report on all considered characteristics. Multiple analysis of variance was used to examine differences in each of the well-being measures among the designated groups over time. SPSS version 24 was used to analyze the data.

## Results

### Study Population Characteristics

Approximately 29.8% of admissions to LTC facilities in the study period had a diagnosis of dementia, 2.9% with schizophrenia, 4.3% with a mental health disorder, and 1.0% with an IDD.

Table 1 shows personal and admission characteristics. Notably, those with schizophrenia and IDD were much younger compared to those with dementia or other mental health diagnoses. Both of these diagnostic subgroups had the largest number of residents <65 years old (schizophrenia: 66.0% and IDD: 57.5%), whereas in the group with dementia, only 4.5% were under 65 years.

**Table 1:** Study population characteristics overall and by diagnostic group.

	**All admissions** ***N* = 1,093,890**	**Subgroups**				
		**Schizophrenia**	**Mental health**	**ID**	**Dementia**	**All others**
		***N* = 31,723**	***N* = 47,037**	***N* = 10,939**	***N* = 325,979**	***N* = 678,212**
**Mean age in years (sd)**	76.2 (14.5)	61.9 (14.1)	75.7 (14.0)	56.6 (19.2)	83.2 (9.2)	74.1 (14.8)
**Gender**						
Male	38.3%	48.8%	40.7%	50.4%	33.8%	39.4%
Female	61.7%	51.2%	59.3%	49.6%	66.2%	60.6%
**Mean depression score (sd)**						
Baseline	3.1 (4.0)	3.3 (4.3)	3.3 (4.3)	2.6 (3.9)	3.1 (4.0)	3.1 (4.1)
3-months	2.5 (3.8)	2.9 (4.1)	2.9 (4.1)	2.2 (3.7)	2.7 (3.8)	2.3 (3.5)
6-months	2.4 (3.6)	2.7 (4.0)	2.7 (3.9)	1.9 (3.4)	2.7 (3.9)	2.3 (3.5)
**Pain**						
Baseline	56.5%	43.7%	43.9%	53.3%	41.6%	63.9%
3-months	47.6%	38.1%	39.2%	48.2%	35.9%	53.6%
6-months	42.7%	32.9%	38.1%	43.4%	35.9%	47.0%
**Behavior**						
Baseline	11.7%	21.3%	32.8%	17.3%	23.5%	5.1%
3-months	12.6%	22.9%	29.3%	19.3%	23.0%	6.1%
6-months	13.2%	23.5%	25.7%	18.9%	20.5%	7.9%

While 61.7% of all residents were female, the percent was much lower for the schizophrenia and IDD cohort—51 and 50% respectively. In terms of marital status, 16.5% of residents had never been married, while a majority of those with schizophrenia and IDD had never been married (54.5 and 74.1%, respectively).

### Mental Wellbeing: Depression

**Figure 1:**
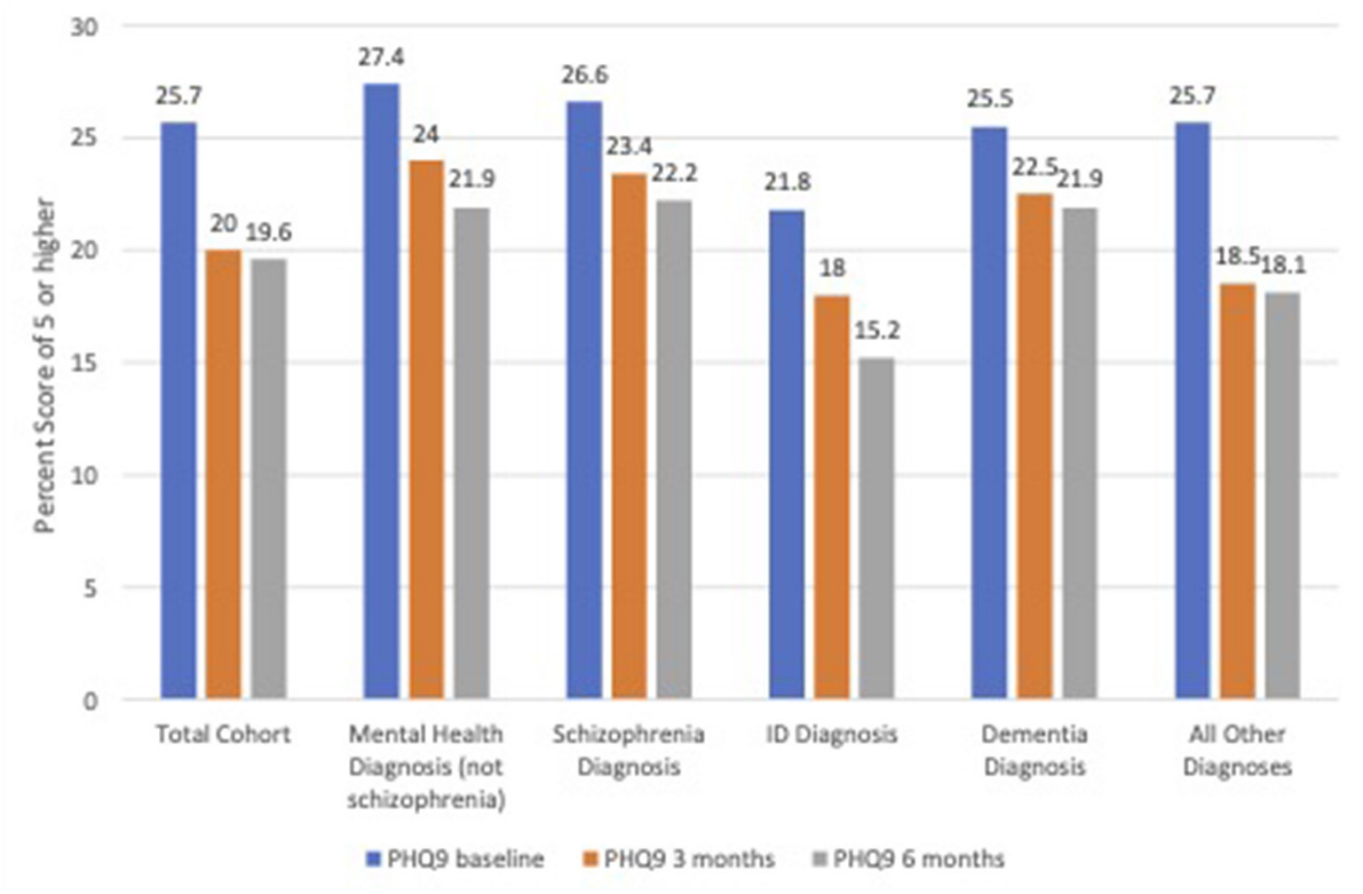
Percent of residents with PHQ-9 score of five or greater at follow-up.

Table 1 shows the mean PHQ 9 scores over time and by group. Overall, average scores reflected a low level of depression at baseline; residents with IDD had the lowest average depression score, while those with schizophrenia and mental health diagnoses had the highest mean scores. Improvements were noted over time in all subgroups, with the largest improvement in the “all others” group and the smallest among those with dementia. Creating of dichotomy of those with no depression (scores 0–4) and those with depression (scores five or greater), Figure 1 displays the percent of residents with depression for the three assessments. The percentage of those assessed as depressed declined over time, with greatest decline in the “all other diagnoses” category.

### Physical Wellbeing: Pain

**Figure 2:**
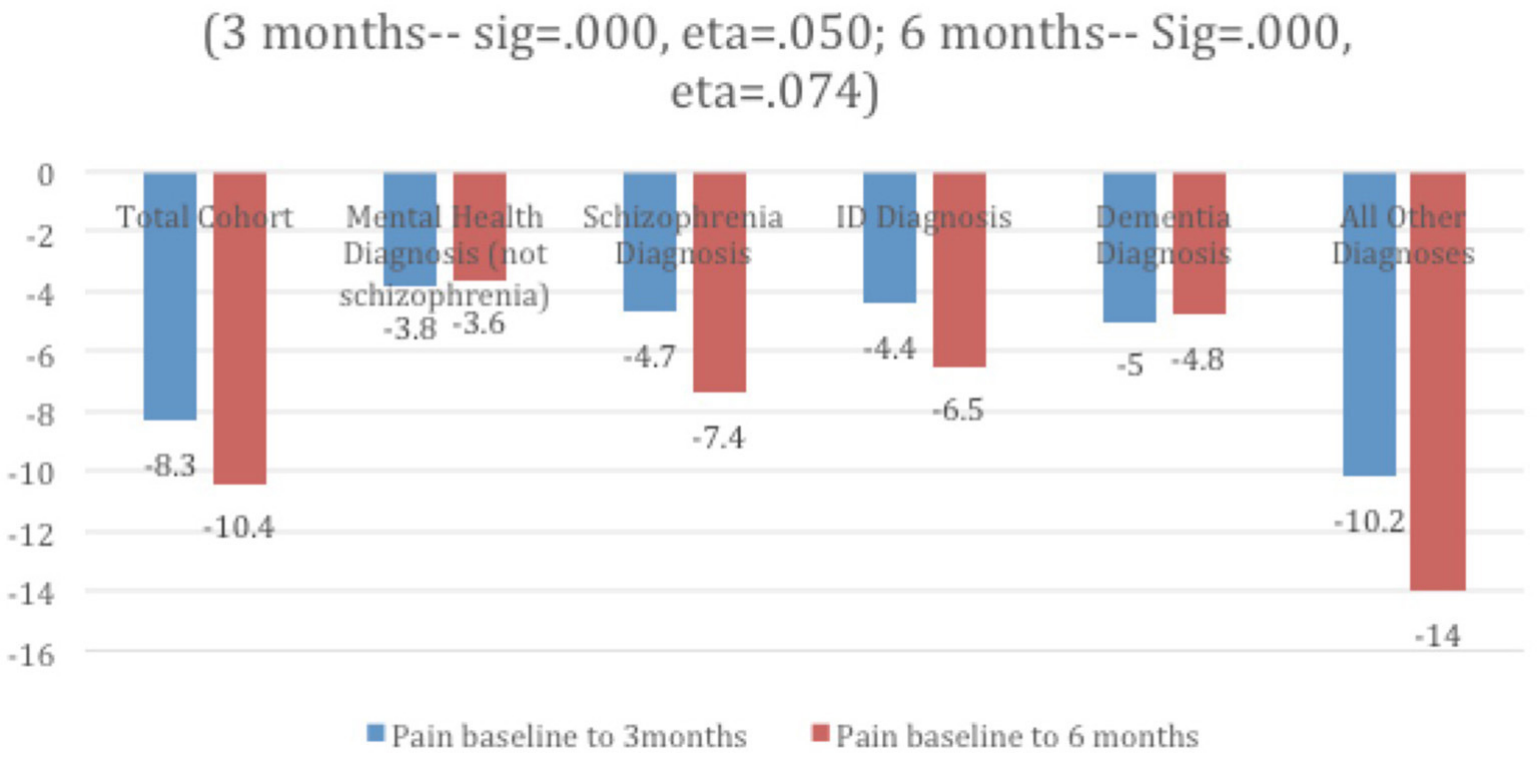
Mean decline in pain % over time and by diagnostic group.

The percentage of residents with pain across diagnostic groups and follow up periods are presented in Table 1. Those with a dementia diagnosis had the lowest percentage presenting with pain at baseline and at 3 month follow-up, while those with schizophrenia had the lowest levels of pain at 6 month follow-up. Improvements were noted over time in all subgroups, with the largest improvement in the “all others” group and the smallest among those with a mental health diagnosis (Figure 2).

### Social Wellbeing: Behaviors

**Figure 3:**
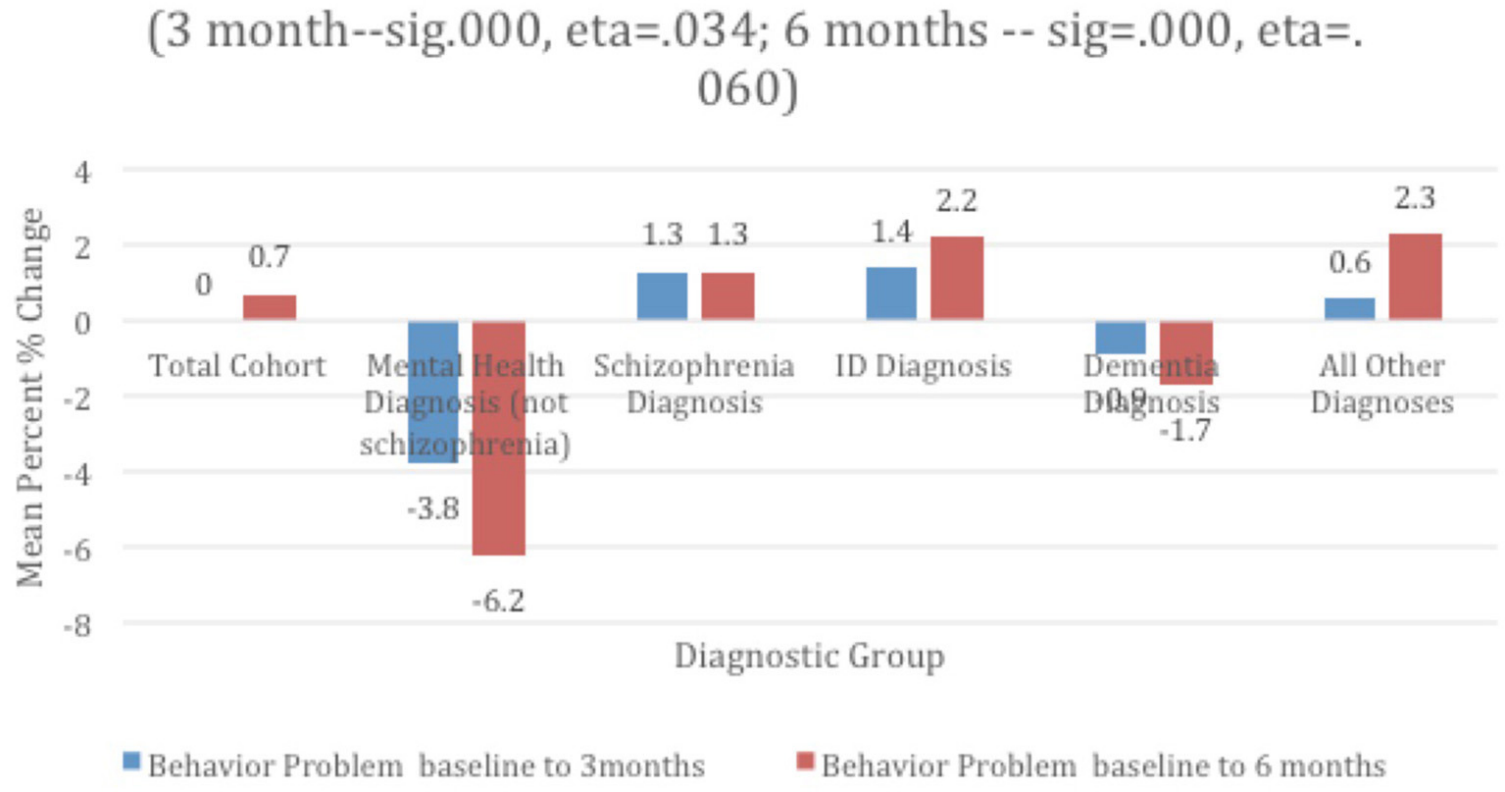
Mean % change in problem behavior over time and by diagnostic group.

Table 1 displays the percentage of persons in the total group and within each subgroup manifesting any one of the behaviors identified as behaviors that challenge. Those with a mental health diagnosis had the largest percentage exhibiting behaviors that challenge at baseline and each follow-up, and it was consistently lowest among the “all others” group. Marked improvements were noted in the group with mental health diagnosis; those in the dementia group also improved over time, but not by as much. Behavior worsened over time for those with schizophrenia and IDD, as well as in the “all others” group. In particular, those with IDD experienced the highest level of worsening at 3 month follow-up and continued to worsen (Figure 3).

## Discussion

This paper examined the extent to which the person-centered care model, that is supposed to underly nursing homes in the United States, is able to improve the individual wellbeing of residents with SMI, IDD, and dementia as well as it does for other residents without such diagnoses. The answer is yes, mostly, and for most.

All study sample LTC residents, regardless of their subgroup membership, experienced improvements in pain and depression, though the group without diagnoses (i.e., “all others”) experienced the greatest gain in both areas. Among the four considered diagnostic groups, those with IDD experienced the largest improvement in depression, while those with dementia experienced lesser levels of improvement in this area over both follow-up periods. The proportion of improvement in pain was similar across the four diagnostic groups at the 3 month follow-up, but varied considerably at 6 months. Here, those with schizophrenia and IDD had the greatest improvement in pain overall; the improvement experienced by those with schizophrenia almost doubled that of persons with mental health disorders. Those with mental health disorders saw the least improvement in pain over time. The pain outcome has to be interpreted with some caution. In a recent Canadian study, a large proportion of long-term care residents with mental and cognitive disorders either did not report pain or reported pain less than daily (13). The challenge of identifying pain among adults with intellectual and developmental disabilities has also been reported (60). The chance for underreporting of pain also may exist in the study population reported here, although we cannot confirm such a bias.

With respect to behaviors that challenge, these were most prevalent among persons with mental health disorders and dementia at baseline and follow-up. These groups were also the only ones to experience overall improvement in behavior over time. It should be noted that the proportion of improvement of those with mental health disorders greatly exceeded that seen among persons with dementia. Behaviors worsened in all other groups. Persons without diagnoses least commonly exhibited behaviors that challenge, and experienced a high level of decline by 6 months. This level of decline was also experienced by persons with IDD, who had the highest rates of worsening overall. At the same time, the rates are much lower, and a lingering effect of both public and self-stigma may be an influencing factor as they contribute to fear, reluctance for social interaction, shame and avoidance (22).

So, it appears that the care provision model in US nursing homes provides the necessary foundation for staff to address depression and pain in persons with complex needs, but less so for behaviors that challenge. Although, when there is an improvement of note, it is for those in the mental health diagnostic group; in this respect, the groups with SMI and IDD did not underperform compared to other groups.

There are a number of possible reasons supporting this outcome. It is possible that LTC staff, most often clinicians, have adequate knowledge and skills to assess and treat depression and pain—two common conditions among older adults. The origins and ways of supporting people with behaviors that challenge may be less straightforward. That said, staff have some better experience with some behaviors like wandering, as it is prevalent among those with dementia. LTC homes take wandering into account when designing facilities, and have appropriate strategies in place to monitor and manage wandering (e.g., alarms, locked doors). As seen in the results, persons with dementia were among the two groups who saw improvements in behavior over time.

We should also note that LTC staff may have less experience with behaviors that are more common among persons with schizophrenia and those with IDD, such as self-injury, socially inappropriate, and destructive behaviors, and they may have received less training in how to recognize—and prevent conditions that lead to such behaviors. Future work is needed to explore the different forms of behaviors that challenge seen in LTC and to determine which behaviors in particular, should be the focus of additional attention to promote wellbeing and quality of life. While both diagnostic groups represent a relatively small proportion of LTC admissions, they are admitted at much younger ages and therefore may have an extended length of stay compared to other residents. There is therefore impetus to understand how LTC staff may better support their needs to prevent or reduce such behaviors. Note, this is an issue for those with dementia as well, thus suggesting that the lack of an approach to address behaviors is not limited to groups with IDD and schizophrenia.

The work presented here was a secondary data analysis and, as such, we were unable to dictate specific data collection elements or scales. We used response categories and scales as they existed in the MDS. We focused on the primary diagnosis recorded during the baseline assessment and did not consider individuals who may have had multiple diagnoses.

## Conclusion

The use of a person-centered model of care in US LTC has been mandated for more than 30 years. This study showed that staff in these settings are able to provide for the mental and physical wellbeing with respect to depression and pain, and this is true even for those with complex needs—defined in this study as persons with schizophrenia, psychotic disorders, and IDD. There remains, however, room for improvement with regard to social well-being and minimizing the occurrence of behaviors that challenge among persons with IDD and schizophrenia. Given the movement away from segregated, specialized institutions and toward use of community-based supports and services, increasing numbers of persons with such diagnoses are being admitted to LTC. Adults with schizophrenia and IDD are admitted at much earlier ages than those without such diagnoses—as many as 20 years earlier, on average. Consequently, more attention to how best to support them is warranted, and in fact, mandated.

## Data Availability Statement

The original contributions presented in the study are included in the article/supplementary material, further inquiries can be directed to the corresponding author.

## Ethics Statement

The studies involving human participants were reviewed and approved by Hebrew SeniorLife, the Marcus Institute for Aging Research. Written informed consent for participation was not required for this study in accordance with the national legislation and the institutional requirements.

## Author Contributions

EH drafted the initial version of the manuscript, with major contributions by LM. JM conducted the analyses and provided major contributions to the writing. GAH provided valuable and important feedback on the manuscript prior to its completion. All authors contributed to the development of the study topic and design.

## Funding

GAH received salary support from the Schlegel Research Chair in Geriatric Medicine.

## Conflict of Interest

The authors declare that the research was conducted in the absence of any commercial or financial relationships that could be construed as a potential conflict of interest.

## Publisher's Note

All claims expressed in this article are solely those of the authors and do not necessarily represent those of their affiliated organizations, or those of the publisher, the editors and the reviewers. Any product that may be evaluated in this article, or claim that may be made by its manufacturer, is not guaranteed or endorsed by the publisher.
